# Genetic Control of Gut Microbial Diversity Enhances Host Resistance to Pathogenic Infections in *C. elegans*

**DOI:** 10.3390/microorganisms14030551

**Published:** 2026-02-27

**Authors:** Rahat Ullah Khan, Boyang Zhang, Hengcheng Liu, Wenping Wu, Jianqi Yang, Yi-Cheng Ma, Cheng-Gang Zou, Ping Jin

**Affiliations:** 1 State Key Laboratory for Conservation and Utilization of Bio-Resources in Yunnan and Key Laboratory of Industrial Microbial Fermentation Engineering of Yunnan Province, School of Life Sciences, Yunnan University, Kunming 650091, China; rahatullah_rahat@yahoo.com (R.U.K.); zhangby20000929@163.com (B.Z.); 15028632239@163.com (H.L.); 15126694665@163.com (W.W.); mayc@ynu.edu.cn (Y.-C.M.); 2 Faculty of Health and Science, University of Macau, Macau SAR, China; cc21798@um.edu.mo; 3 Southwest United Graduate School, Kunming 650092, China

**Keywords:** *Caenorhabditis elegans*, gut microbes, microbiota–host interaction, mpk-1 mutant, innate immunity

## Abstract

Intestinal bacteria play crucial roles in maintaining host health and regulating disease. While much of the current research has focused on how changes in the gut microbiota affect various physiological functions of the host, little is known about how the host’s genetic factors shape gut microbiota diversity or how gut-dominant bacteria influence host innate immunity and lifespan. In this study, we demonstrated that a mutation in the *Caenorhabditis elegans* ERK-encoding gene, *mpk-1*, promotes the enrichment of *Raoultella planticola* in the gut of worms, and the bacterium confers resistance to infection by the pathogenic bacterium *Pseudomonas aeruginosa* PA14 (PA14) in worms. Mechanistically, a compromised immune response, which is dependent on the *let-60–mpk-1* pathway, promotes the colonization of *R. planticola* in *mpk-1* mutants. Importantly, *R. planticola* induces autophagy, thereby enhancing nematode resistance to PA14 infection and extending its lifespan. Our findings shed light on how immune-compromised *mpk-1* mutants increase colonization permissiveness and utilize *R. planticola* to bolster their antibacterial immunity against pathogenic *P. aeruginosa*, offering new insights into the regulatory mechanisms of host–microbiota interactions. These results emphasize the complex interplay between host genetics, the microbiota, and immune responses, providing potential therapeutic strategies to modulate the microbiota for improved health outcomes.

## 1. Introduction

From nematodes to mammals, microbes colonize nearly every surface of the body, particularly the gastrointestinal tract, where they impact nutrition, tissue development, immune system maturation, pathogen resistance, and potentially behavior [1]. The intestines harbor complex bacterial communities that play crucial roles in maintaining host health and regulating disease. Over the course of evolution, animals and microorganisms have developed intricate, interdependent relationships, with the microbiota significantly influencing host physiology and overall fitness [2]. These microbial communities (Microbiota) form complex networks that interact with host genetic factors, collectively shaping host traits, promoting biodiversity, and maintaining ecological stability [3]. Microbial interactions within the host ecosystem are diverse: some species produce toxins to outcompete others, whereas others secrete enzymes that foster mutual benefits. In turn, hosts have evolved mechanisms to suppress harmful microbes while supporting beneficial strains, driving coevolution and generating stable mutualistic relationships [4]. Understanding these interactions requires categorizing the microbiome into three classes of effects—microbe-to-host, host-to-microbe, and microbe-to-microbe—each with distinct evolutionary characteristics [2]. This framework helps clarify how the microbiota protects against pathogens, promotes the immune response, and supports overall host fitness. A disruption in microbial community structure and/or function (Dysbiosis) will alter host fitness or disease susceptibility. Research has provided compelling evidence of the crucial role of the microbiome in various vital functions of the body, including metabolism, nutrition, immune function, physiology, and disease prevention. However, much remains unknown about how the host’s genetic factors shape gut microbiota diversity and how gut-dominant bacteria affect host innate immunity and lifespan.

Hosts regulate their microbiome through the immune response, barrier function, physiological homeostasis, and microbial transit, shaping microbial traits to benefit the host. This dynamic has led to the concept of the microbiome as “an ecosystem on a leash” where symbionts interact in ecological communities strongly influenced by host control [5]. While the impact of the microbiome on host physiology, including metabolism, immune function, and behavior, is well documented, less is known about the active role of hosts in manipulating microbiomes. Host genetics are estimated to account for approximately 10% of microbiome beta diversity variance, although this variance varies widely [6]. Similar studies, such as one analyzing over 1000 Fecal samples from UK twins, identified *Christensenellaceae* as the most heritable taxon linked to improved metabolism and lower body mass. Understanding the specific mechanisms by which the host influences microbial colonization is crucial for comprehending host-microbiome interactions.

*Caenorhabditis elegans* is a robust model for studying host–microbe interactions because of its reproducible gut microbiota, which overlaps significantly with bacteria from its natural habitats. Its genetic tractability and compatibility with next-generation sequencing allow detailed exploration of bacterial effects on host traits such as stress resistance, longevity, and development. Throughout its life, *C. elegans* encounters pathogenic microorganisms, making resistance to these pathogens crucial for maintaining health and quality of life. The innate immune system of *C. elegans* plays a pivotal role in health span and is regulated by several evolutionarily conserved signaling pathways, such as the ERK MAPK MPK-1 pathway, the mitochondrial stress response pathway transcription factor ATFS-1 [7], the TEAD orthologue EGL-44 [8] pathway, the cell death protease CED-3 [9], the TFEB orthologue HLH-30-mediated autophagy and lysosomal pathway [8], the insulin-like DAF-2/dAF-16 pathway, the endoplasmic reticulum unfolded protein response pathway IRE-1/XBP-1, and the DAF-7/TGF-β-like signaling pathway [9]. We selected mutants in *mpk-1*, *atfs-1*, *egl-44*, *ced-3*, and *hlh-30* because they represent distinct, conserved modules in immune regulation and cellular homeostasis, enabling us to examine how host pathways shape gut community composition and fitness outcomes. Specifically, ERK MAPK MPK-1 supports pathogen defense in part through autophagy; ATFS-1 links mitochondrial stress responses to antimicrobial programs and promotes clearance of PA14; HLH-30 regulates antimicrobial and lysosome-autophagy genes required for host defense; CED-3 controls apoptosis and epithelial homeostasis under infection-associated stress; and ELG-44 regulates immune response–related transcriptional programs. Together, this panel allows systematic testing of pathway-specific effects on colonization and host phenotypes.

In certain cases, hosts can enhance their defense against pathogenic invasion by employing beneficial microorganisms. These intestinal bacteria can significantly extend the nematode lifespan. For example, studies with *Mycobacterium* sp. Root265 identified bioactive molecules, including polysaccharides and arabinogalactan peptidoglycan, which promote longevity via daf-16-dependent and daf-16-independent pathways, highlighting the therapeutic potential of bacterial derivatives [10]. Our laboratory has identified several signaling pathways that mediate resistance to infections, including the ERK MAPK autophagy pathway [11], the YAP-TEAD signaling pathway, and the TOR signaling pathway. These findings highlight the intricate relationships among intestinal bacteria, autophagy, and immune signaling pathways in *C. elegans*, providing insights into host–pathogen interactions. In-depth research into the infection resistance mechanisms of nematodes will increase our understanding of the underlying causes of infection- and immunity-related diseases, providing a theoretical foundation for their prevention and treatment.

This study used *C. elegans* to investigate the impact of host gene mutations on the gut microbiota. L1 larvae of wild-type worms (N2) and five immune-related gene mutants (*mpk-1, atfs-1, egl-44, ced-3*, and *hlh-30*) were exposed to the soil microbiota, followed by full-length 16S rRNA sequencing, which identified 25 phyla and revealed the core microbiota of 144 shared amplicon sequence variants (ASVs). Mutant worms displayed significant differences in bacterial diversity at the genus level, highlighting the role of host genetics in shaping the gut microbiota. *R. planticola*, an environmental Enterobacteriaceae member commonly associated with soil and water niches that can colonize animal hosts under certain conditions, was enriched in mpk-1 mutants and promoted resistance to *P. aeruginosa* PA14 (an opportunistic pathogen that is widely used in *C. elegans* infection models to quantify host defense using survival readouts) by activating bec-1-mediated autophagy, which also extended nematode lifespan. In summary, these findings elucidate the effects of host genetics on the gut microbial composition and the mechanisms by which dominant gut bacteria influence host health and immunity, providing new insights into the regulatory mechanisms of host–microbiota interactions.

## 2. Materials and Methods

### 2.1. Soil Microbiota Preparation and Worm Exposure

Orchard soil, natural forest soil, and garden soil were mixed in equal proportions (1:1:1). Various types of humus, including decomposing fruit, plant stems, and leaves, were added to the mixture, which was then thoroughly blended and allowed to ferment for two weeks. After fermentation, the mixture was divided into two portions. One portion was soaked in ultrapure water for one week to extract microorganisms. The resulting supernatant was collected and centrifuged at 2000 rpm to remove sediment, thereby eliminating protozoa and other small organisms, and enriching the microbial fraction. The second portion was sterilized by autoclaving at 121 °C for 30 min, a process that was repeated to ensure the elimination of most microorganisms. The microbial leachate from the non-sterile soil was then added to the sterilized soil, mixed thoroughly, and allowed to ferment for an additional two weeks. Synchronized *C. elegans* at the L1 larval stage were introduced into the treated soil. The soil was kept moist and monitored over 3–4 days, during which the nematodes colonized the soil and their guts were colonized by the introduced microbial community. Each experiment included three biological replicates. Within a given experiment, the three replicates used the same prepared soil batch; however, independent experiments were conducted using independently prepared soil samples.

### 2.2. Recovery of Wild Nematodes

Soil containing nematodes was divided into small portions and wrapped in multiple layers of filter paper. The edges were sealed, and the packets were placed in a Baermann funnel apparatus to facilitate drainage. Sterile M9 solution was added to the funnel, allowing nematodes to migrate downward and settle at the bottom by gravity. The nematodes were collected overnight into a sealed conical flask. The next day, the flask was removed, and the supernatant was carefully discarded, leaving the concentrated nematodes for subsequent use.

### 2.3. Isolation, Culture and Identification of Intestinal Bacteria of C. elegans

Twenty surface-sterilized *C. elegans* individuals were transferred into a 1.5 mL sterile microcentrifuge tube containing 100 μL of sterile M9 buffer and two sterilized steel grinding balls. The nematodes were homogenized using an automatic tissue grinder, followed by centrifugation at 4000 rpm for 2 min. The supernatant was collected and serially diluted 10-, 100-, 1000-, and 10,000-fold. From each dilution, 100 μL was plated onto sterile 9 cm plates containing brain heart infusion (BHI), lysogeny broth (LB), nutrient agar (NA), and glucopeptone agar (AA). The samples were evenly spread and incubated in two conditions: sealed and unsealed. Plates were placed upside down in a sterile anaerobic incubator at 37 °C. As a control, supernatant from unground wild nematodes was similarly plated. After 6–8 h of incubation, plates were examined for single colonies, ensuring that colonies derived from the grinding solution represented gut-associated bacteria. Individual colonies were picked and cultured in 700 μL of the corresponding liquid medium at 37 °C with shaking. Bacterial identification was performed by amplifying the 16S rRNA gene using primers 16S-27F and 16S-1492R, followed by sequencing. Based on sequence analysis, redundant strains were consolidated. The unique isolates were then cultured and preserved for future use.

### 2.4. 16S rRNA Sequencing and Microbial Community Analysis

Recovered nematodes were transferred into sterile 1.5 mL microcentrifuge tubes, with 300–500 μL of nematode suspension added per tube. The tubes were securely sealed, rapidly frozen in liquid nitrogen, and submitted for 16S rRNA gene sequencing. Sequencing was performed by Shanghai Meiji Biomedical Technology Co., Ltd., Shanghai, China, using primers 27F and 1492R to amplify the full-length 16S rRNA gene on the Illumina sequencing platform. Subsequent data processing and species annotation were conducted using the laboratory’s cloud-based platform, incorporating the SILVA database and R Studio (R 4.3.3) for analysis.

Sequence data were processed using QIIME2(2024.5), including quality filtering, merging, and denoising to infer ASVs with chimera removal. Taxonomic classification was conducted using the SILVA reference database. Statistical analyses included the Kruskal–Wallis test and alpha diversity metrics (Chao1, ACE, Simpson, and Shannon indices) to compare bacterial diversity across different mutant strains. Beta diversity was evaluated using principal coordinate analysis (PCoA) based on Bray–Curtis distances, revealing distinct microbial community clustering among experimental groups. Differentially abundant bacterial genera were identified using linear discriminant analysis effect size (LEfSe). Samples from different genotypes within the same experiment were processed and sequenced together, and sample processing order was randomized across genotypes. Potential batch effects were evaluated by inspecting ordination patterns stratified by sequencing run/library preparation and were not found to dominate sample clustering. We performed additional sequencing-depth normalization prior to diversity analyses.

### 2.5. Culturing Gut Bacteria from C. elegans

Wild-type (*C. elegans* N2) and mutant strains (*mpk-1*, *atfs-1*, *egl-44*, *ced-3*, and *hlh-30*) were subjected to surface sterilization, and their intestinal bacteria were isolated and cultured. Homogenized nematode suspensions were evenly spread onto four different agar media: AA, LB, NA, or BHI. The plates were incubated at 37 °C to allow bacterial growth. Each medium supported the development of numerous distinct single colonies. Colonies exhibiting diverse morphological characteristics were selected, isolated, and purified. Purified strains were identified through 16S rRNA gene sequencing following PCR amplification. The resulting sequences were analyzed using EzBioCloud’s nucleotide alignment tool (BLASTn (2.14.1)) and compared against reference sequences in the GenBank 16S rRNA gene database for species identification. A complete list of bacterial isolates/strains used in survival screening and mechanistic assays is provided in the Supplementary Materials.

### 2.6. RNA Interference

Bacterial strains were retrieved from an ultra-low temperature freezer, kept on ice, and transferred to a UV-sterilized clean bench. The lid of the 96-well plate was carefully removed, and the protective film was peeled back to locate the desired bacterial strain. Using a sterile tool, a small amount of bacteria was picked and streaked in an S-shape onto a sterile LB agar plate containing 100 μg/mL ampicillin. The plate was sealed and incubated at 37 °C for 8–12 h until single colonies appeared. A single colony was then inoculated into sterile LB liquid medium supplemented with ampicillin and cultured at 37 °C with shaking at 180 rpm for 15–18 h. Subsequently, NGM-IPTG plates seeded with the RNAi-expressing bacteria were incubated upside down at 25 °C for 16–20 h to induce RNA interference. Synchronized L1-stage *C. elegans* were transferred onto these RNAi plates under sterile conditions and incubated at 20 °C. For RNAi-based comparisons, N2 animals fed with the empty vector (EV) were used as the control. After approximately 40 h, the worms reached the young adult stage, and RNA interference was considered complete.

### 2.7. Lifespan Analysis of C. elegans

L1-stage nematodes were transferred onto 9 cm NGM plates seeded with bacteria (Cultured gut isolates) under sterile conditions in a super-clean bench and incubated at 20 °C for 40 h until they reached the young adult stage. The nematodes were then washed with sterile M9 buffer in a clean environment and transferred to 1.5 mL sterile centrifuge tubes, where they were allowed to settle by gravity. The supernatant was carefully removed, and the washing process was repeated 3–5 times until the suspension appeared clear. The cleaned nematodes were then transferred onto 6 cm NGM plates seeded with bacteria, with approximately 30–50 nematodes per plate. Lifespan analysis was subsequently conducted, and survival curves were generated using GraphPad Prism (9.5).

### 2.8. Infection of C. elegans with P. aeruginosa PA14

The *P. aeruginosa* PA14 strain was cultured in LB liquid medium. A total of 300 μL of the bacterial culture was transferred to a sterile 6 cm NGM-FUDR plate under aseptic conditions and evenly spread across the surface. Excess liquid was removed, and the plate was air-dried, sealed, inverted, and incubated at 37 °C for 12 h until the medium was fully covered with bacteria and exhibited characteristic green pyocyanin pigmentation. The plate was then incubated inverted at 25 °C for an additional 12 h. Adult *C. elegans*, previously cultured on 9 cm NGM plates with bacteria at 20 °C for 48 h, were harvested under sterile conditions. The worms were washed with sterile M9 buffer and transferred to 1.5 mL sterile centrifuge tubes to settle naturally. The washing step was repeated 3–5 times until the supernatant was clear. The cleaned nematodes were then transferred onto the prepared *P. aeruginosa* PA14 infection plates, with approximately 50–60 nematodes per plate. Each experimental group included 3–5 plates. Plates were incubated upright at 25 °C, and worm survival was monitored every 12 h. The number of dead worms was recorded until all individuals had died. Survival data were compiled, and Kaplan–Meier survival curves were generated using GraphPad Prism, with each group comprising three replicate plates.

### 2.9. Colony Forming Unit (CFU) Bioassay

*R. planticola* was cultured overnight in BHI broth at 37 °C with shaking, then centrifuged to concentrate the bacterial suspension to the desired density for feeding. Standard NGM plates were prepared, allowed to solidify, and subsequently seeded with *R. planticola*. Under sterile conditions, synchronized L1-stage *C. elegans* (both mpk-1 mutants and N2 wild-type strain) were transferred onto 9 cm NGM plates seeded with *R. planticola* and incubated at 20 °C for 48, 66, and 96 h. After incubation, the worms were washed with sterile M9 buffer and transferred to 1.5 mL sterile centrifuge tubes to settle by gravity. The supernatant was carefully removed, and the washing step was repeated 3–5 times until the suspension appeared clear. The cleaned worms were then homogenized to release gut-associated bacteria. The resulting lysates were serially diluted and plated onto various types of agar media. Plates were incubated overnight at 37 °C, and colony-forming units (CFUs) were counted to quantify bacterial load. Data were analyzed and visualized using GraphPad Prism.

### 2.10. Autophagy Bioassay Using E. coli OP50 and R. planticola

*R. planticola* was cultured in BHI liquid medium, and *Escherichia coli* OP50 was grown in LB. A 200 μL aliquot of each bacterial culture was transferred onto separate sterile 6 cm NGM plates under aseptic conditions. The plates were inverted and incubated at 25 °C for over 16 h to allow for bacterial lawn formation. Synchronized L1-stage LGG-1::GFP transgenic *C. elegans* were transferred to 9 cm NGM plates seeded with either *R. planticola* or *E. coli* OP50 and incubated at 20 °C for 36 h until the worms reached the early young adult stage. The worms were then washed with sterile M9 buffer and transferred to 1.5 mL sterile centrifuge tubes. After settling, the supernatant was removed, and the washing process was repeated 3–5 times until the solution became clear. To immobilize the worms, a small volume of 10 mM levamisole in M9 buffer was added. A drop of the worm suspension was then placed on a microscope slide, covered with a coverslip, and examined under a fluorescence microscope equipped with GFP filters. Fluorescence imaging focused primarily on intestinal cells to evaluate autophagy activity. Images were captured for 30–50 worms per group, and autophagy levels between treatment and control groups were quantified and compared using ImageJ (1.54g) and GraphPad Prism (9.5).

### 2.11. Brood Size Bioassay

L4-stage *C. elegans* were transferred to NGM plates seeded with either *Raoultella planticola* (RP) or *E. coli* OP50 as the food source and maintained at 20 °C. Brood size was assessed daily over a five-day period, beginning on the first day of adulthood (day 1). To prevent overcrowding and cross-contamination, each worm was transferred to a fresh plate every day. The total number of eggs laid by each worm was recorded daily, with a minimum of 10 worms included per treatment group. The assay was performed in biological triplicates to ensure the robustness and reproducibility of the data.

### 2.12. RNA-Seq and Transcriptome Analysis

*E. coli* OP50 and *R. planticola* were cultured overnight at 37 °C with shaking in LB and BHI broth, respectively, and then centrifuged to concentrate the bacterial cells for nematode feeding. The let-60 RNAi bacterial strain was cultured in LB containing ampicillin and tetracycline, and RNAi induction was performed by adding 1 mM IPTG and incubating at 37 °C for 4 h. Standard NGM plates were prepared and seeded with *E. coli* OP50, *R. planticola*, or a 1:1 mixture of *let-60* RNAi bacteria and *R. planticola*. These plates were incubated at 25 °C for 16–20 h to allow bacterial lawn formation. Synchronized N2 strain *C. elegans* eggs were hatched and grown to the L1 larval stage in M9 buffer before being transferred onto the prepared NGM plates. For RNAi experiments, L1 larvae were placed on plates containing the *let-60* RNAi/*R. planticola* mixture and incubated at 20 °C for 42 h, and the control group was EV-fed N2. Following this, the worms were thoroughly washed with M9 buffer to remove residual bacteria and transferred to plates seeded with *Pseudomonas aeruginosa* PA14 for 24 h of exposure. After the infection period, worms were collected by washing them off the plates with M9 buffer, and cleaned nematodes were transferred into sterile microcentrifuge tubes. The samples were flash-frozen in liquid nitrogen and stored at −80 °C for subsequent transcriptomic analysis.

### 2.13. Statistical Analysis

All experiments were performed with at least three independent biological replicates. Data were analyzed using GraphPad Prism (9.5) and R 4.3.3. For comparisons between two groups, two-tailed unpaired Student’s *t*-tests were used when data met assumptions of normality and homogeneity of variance. For comparisons among multiple groups, one-way ANOVA with Tukey’s post hoc test (parametric) or Kruskal–Wallis test with Dunn’s multiple-comparison correction (nonparametric) was used, as appropriate. Survival data from PA14 infection and lifespan assays were analyzed using Kaplan–Meier survival curves and compared using the log-rank (Mantel–Cox) test. For microbiome community analyses, beta-diversity was calculated using Bray–Curtis distances and visualized by PCoA/UMAP. Unless indicated otherwise, results are presented as mean ± SEM. Multiple-testing correction was applied where relevant (e.g., LEfSe or transcriptomic enrichment analyses). A *p* < 0.05 was considered statistically significant.

## 3. Results

### 3.1. The Similarity and Variation in Microbial Community Structures Across Soil, N2 and Five Mutant Strains of Immune-Related Genes

To investigate the microbial community in the intestine of *C. elegans*, we introduced sterile L1 larvae of the N2 wild-type strain and five immune-related gene mutants (*mpk-1*, *atfs-1*, *egl-44*, *ced-3*, and *hlh-30*) into the soil. These worms were cultured for 3 to 4 days until they matured into adults. After both the soil and worm samples were collected, we extracted DNA from the soil and from the six worm types to analyze the full-length bacterial 16S rRNA genes. A total of 913,710 reads were generated from 70 samples via barcode-based identification. Each sample generated a minimum of 8293 and an average of 13,053 circular consensus sequencing (CCS) reads. We identified 25 phyla, 49 classes, 123 orders, 227 families, and 533 genera. At the family level, the microbiota in the soil, the N2 strain, and the different mutants showed notable differences. In the soil, the most dominant families were *Xanthomonadaceae*, *Enterobacteriaceae*, and *Rhizobiaceae*. Among the N2 strain and mutants, the most dominant families were *Enterobacteriaceae*, *Yersiniaceae*, *Lactobacillaceae* and *Weeksellaceae* (Figure S1A). At the genus level, a total of 533 genera were annotated, with variations across the groups. The relative abundances of genera varied between the wild-type N2 and mutant strains and were distinct from those of the soil microbiota at the genus level (Figure S1B). However, the mutants presented high similarity to the N2 strain, with only minor differences at the genus level.

### 3.2. Variation and Diversity of the Gut Microbiome in Different Groups

**Figure 1:**
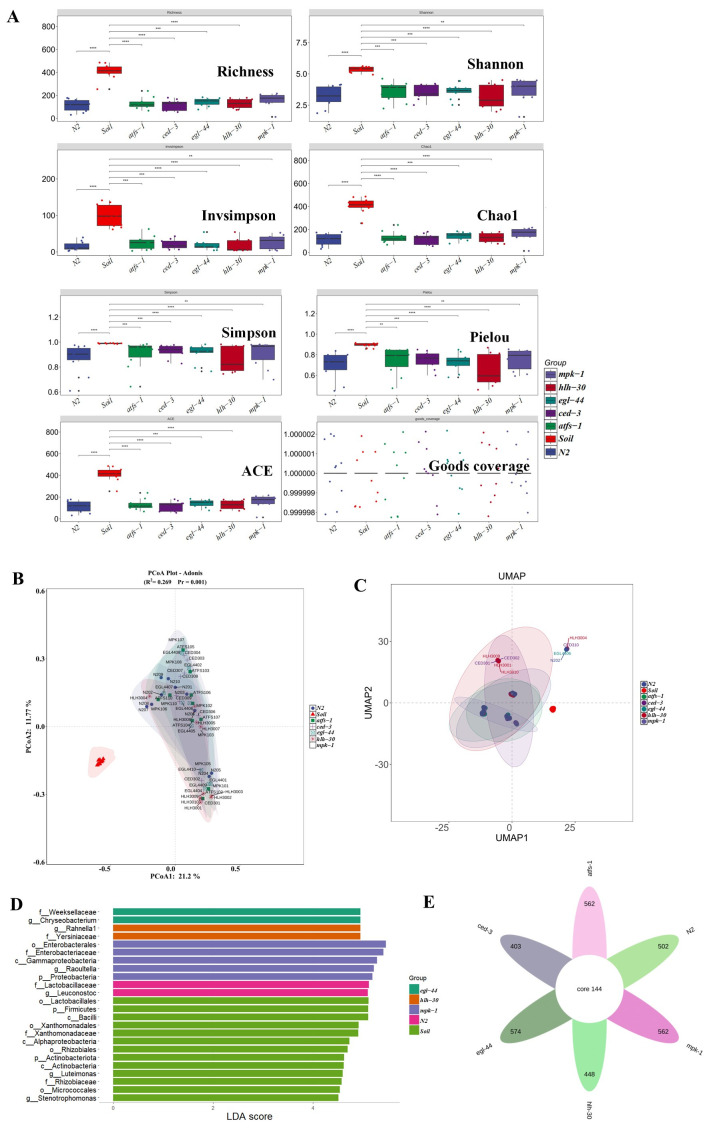
Variation and diversity of the gut microbiome across soil, N2 and five mutant strains. (**A**) Different indices of alpha diversity. The values represent the means ± standard deviation (*n* = 10). N2 strain: wild-type worms; *mpk-1*, *egl-44*, *atfs-1*, *ced-3* and *hlh-30* are immune-related gene mutants. (**B**) The Bray–Curtis distance is the basis for principal coordinate analysis (PCoA) of each group. (**C**) UMAP analysis revealed distinct clustering of microbial communities between the groups. (**D**) Lefse analysis (LDA bar chart) of the bacterial communities in the soil, N2 strain and five mutants. LDA score = 4.5. (**E**) Venn diagram analysis of the different groups. The data are shown as the means  ±  s.d.; **, *p* < 0.01; ***, *p* < 0.001; ****, *p* < 0.0001. NA, not applicable.

Several alpha diversity metrics, including richness, the Shannon index, Invsimpson, Chao1, Simpson, Pielou, the abundance-based coverage estimator (ACE), and Good’s coverage, were employed to investigate the number of different ASV features (i.e., species richness) between different groups (Figure 1A, Supplementary Figure S1C,D). The overall results suggest that the soil samples present the greatest microbial diversity and richness. Among the five mutant strains, *mpk-1* mutants showed the highest values for the S.obs (the number of observed features), Chao1, ACE, Shannon, and Pielou indices, reflecting the richest and most diverse microbial communities. In contrast, *hlh-30* mutants consistently displayed the lowest diversity across most indices. The highest Simpson index was observed in *ced-3* mutants, while *hlh-30* again showed the lowest. These results indicate that the genetic mutations in these mutant strains may impact the gut microbiome, leading to altered diversity compared with that of the wild-type. Furthermore, principal coordinate analysis (PCoA) and uniform manifold approximation and projection analysis (UMAP) revealed distinct clustering of microbial communities between the groups, with significant separation among the N2, soil, and mutant strains (Figure 1B,C). The N2 group and soil samples presented the most distinct clusters, whereas the mutant strains presented a more diverse pattern but with some overlap in their microbial composition. A greater distance indicates greater dissimilarity, indicating that the microbial communities in the gut of the mutants are quite distinct from those found in their natural soil habitat. LEfSe revealed that the largest number of taxonomic biomarker groups were detected in the soil samples, followed by the mpk-1 mutant and then the *egl-44* and *hlh-30* mutants (Figure 1D), suggesting that specific gut microbiota features are related to different groups. The number of ASVs found in the *egl-44* mutants was 718, followed by the *atfs-1* and *mpk-1* mutants, which had 706, followed by the N2 strain with 646 and the *hlh-30* and *ced-3* mutants, respectively. Analysis of the core microbiome revealed 144 shared ASVs across all groups, with a significant number of unique ASVs observed in the individual mutant strains (*mpk-1*, *egl-44*, and *egl-3*). Specifically, *egl-44* and *mpk-1* presented the greatest number of unique ASVs (574 and 562, respectively), indicating strain-specific microbiome signatures (Figure 1E). Together, these results demonstrate that the gut microbiome composition and diversity are significantly altered across different mutant strains and soil samples, with notable differences in richness, diversity, and community structure, particularly in the *mpk-1* mutant strain. These differences underscore the importance of host genetics in shaping the microbial communities in *C. elegans* and further suggest potential implications for understanding immune system regulation and gut microbiome dynamics.

### 3.3. Intestinal Bacteria Confer Resistance to Pseudomonas aeruginosa PA14 Infection in Nematodes

**Figure 2:**
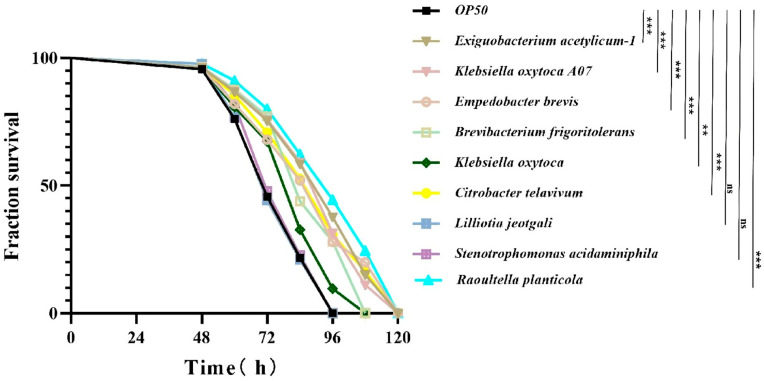
Cultured bacteria confer *C. elegans* resistance to *P. aeruginosa* PA14. *Exiguobacterium acetylicum*, *Klebsiella oxytoca A07*, *Empedobacter brevis*, *Brevibacterium frigoritolerans*, *Klebsiella oxytoca*, *Citrobacter telavivum* and *R. planticola* increased the survival of N2 worms exposed to PA14. *Lilliotia jeotgali* and *Stenotrophomonas acidaminiphila* did not influence the survival of N2 worms exposed to PA14. The data are shown as the means  ±  s.d. (*n* = 3 biological replicates, one-way ANOVA); **, *p* < 0.01; ***, *p* < 0.001. ns, not significant.

Since microbial symbionts often protect against pathogens [12], we next investigated the impact of these dominant bacteria on the host. To capture bacteria in worms, we cultured bacteria and isolated 33 species of bacteria from the guts of nematodes. The phylogenetic tree revealed that 33 strains of intestinal bacteria belong to 3 phyla: *Proteobacteria*, *Bacteroidetes* and *Firmicutes*. These strains were further categorized into five classes, *Betaproteobacteria*, *Gammaproteobacteria*, *Flavobacteria*, *Bacilli* and *Actinobacteria*, which belong to 7 orders and 10 families (Figure S2A). We tested nine species of bacteria, and found that six of these bacteria, *Exiguobacterium acetylicum*, *Klebsiella oxytoca A07*, *Empedobacter brevis*, *Brevibacterium frigoriterans*, *Klebsiella oxytoca*, *Citrobacter telavivum*, and *R. planticola* (Figure 2), significantly improved *C. elegans* survival compared with the control strain OP50, with enhanced resistance to *P. aeruginosa* PA14. On the other hand, some bacteria, such as *Stenotrophomonas acidaminiphila* and *Liliotia jeotgali* (Figure 2), did not significantly affect survival, indicating variability in bacterial effectiveness.

We next analyzed the abundance of these bacteria in the 16S sequencing data, and the results revealed that these bacteria were relatively enriched in different groups. Notably, *R. planticola* was significantly more abundant in the *mpk-1* mutants than in the other strains, with the highest abundance observed in the *mpk-1* mutants (*p* < 0.05) (Figure S2B). In contrast, the abundance of *Klebsiella oxytoca* did not significantly differ between the mutant strains, with relatively consistent abundances across the atfs-1, egl-44, and *hlh-30* mutants, although the *ced-3* mutants presented slightly lower abundances (Figure S2C). The abundance of *Empedobacter brevis* was significantly greater in the N2 strain than in the mutants, particularly the *mpk-1* and *hlh-30* mutants, whose abundance was much lower (Figure S2D). *Brevivacterium frigoriterans* presented the most pronounced differences in abundance (Figure S2E). These findings highlight significant variation in the abundance of specific gut bacteria across different *C. elegans* strains and mutants, suggesting the influence of host genetic factors on microbiota composition. Certain bacterial species, such as *R. planticola*, may be more prominent in specific mutants (such as *mpk-1*), potentially influencing their physiological traits and immune response.

### 3.4. R. planticola as a Significant Biomarker in mpk-1 Mutant Strains

**Figure 3:**
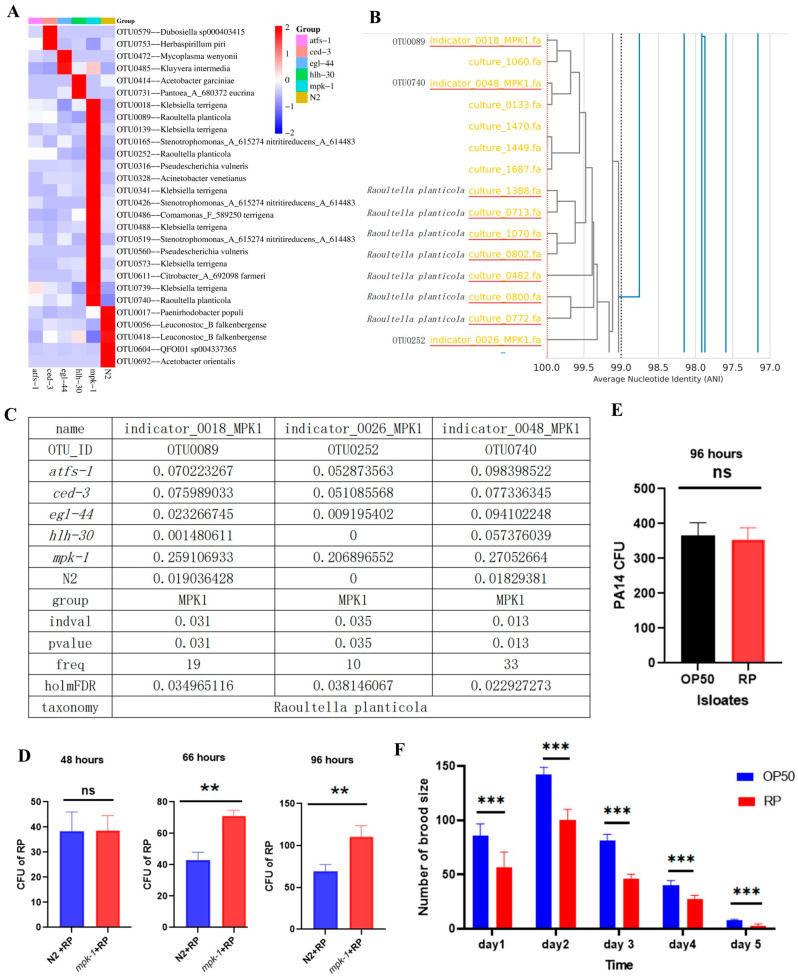
*Raoultella planticola* as a biomarker in *mpk-1* mutants. (**A**) Heatmap of biomarkers at the ASV level in soil, the N2 strain and these mutants *(mpk-1*, *ced-3*, *atfs-1*, *hlh-30* and *egl-44*). The red line indicates *R. planticola* at the OTU level as a significant biomarker in *mpk-1* mutants. (**B**) ANI tree files constructed from cultured strains and *mpk-1* indicator bacteria. The ANI between the cultured strains and the indicator bacteria was greater than 95% in all cases. (**C**) Indicator species analysis results showing that OTU0089, OTU0252 and OTU0740 are indicator bacteria of *mpk-1.* The tree files represent indicator-0018, indicator-0026 and indicator-0048. (**D**) *R. planticola* colonizes and proliferates in *mpk-1* mutants over time. (*n* = 3 for each group, Student’s *t*-test). (**E**) *R. planticola* cannot influence the load of the pathogenic bacteria PA14 in the intestine of *C. elegans*. (*n* = 3 for each group, Student’s *t*-test). (**F**) Impact of *R. planticola* on the brood size of worms. (*n* = 3 for each group, Two-way ANOVA). The data are shown as the means  ±  s.d. **, *p* < 0.01; ***, *p* < 0.001. ns, not significant.

Specific gut microbiota features can reflect specific diseases or normal physiological conditions in the host. The distribution analysis across groups identified distinct amplicon sequence variants (ASVs) that were significantly enriched in specific mutant backgrounds. Specifically, the *mpk-1* mutant exhibited a particular focus on *R. planticola* at the ASV level (Figure 3A and Figure S3). To further confirm the presence of *R. planticola* as a prominent biomarker in the mpk-1 mutant group, we performed ANI tree analysis to compare genetic similarity between cultured strains and the indicator bacteria identified in mpk-1 mutants. Notably, cultured *R. planticola* exhibited greater than 99.5% average nucleotide identity with the recorded indicator of mpk-1 mutants, confirming that the bacterial strains associated with mpk-1 are closely related to *R. planticola* (Figure 3B,C). This high level of genetic similarity underscores the reliability of identifying *R. planticola* as a significant microbiome feature in mpk-1 mutants, highlighting its potential role in the altered gut microbiome associated with this mpk-1 mutant strain.

As mentioned above, the level of *R. planticola* was most abundant in the mpk-1 mutants according to 16S rRNA sequencing. We therefore evaluated the colony-forming units (CFUs) of *R. planticola* in the wild-type (N2) strain and mpk-1 mutant *C. elegans* over different time periods. Quantitative analysis of *R. planticola* revealed that, compared with N2 worms, mpk-1 mutants presented significantly greater CFUs of *R. planticola* at 66 and 96 h post-infection, suggesting an increased bacterial load in these mutants (Figure 3D). However, no significant difference was observed at 48 h (p = ns). These findings suggest that the presence of *R. planticola* is correlated with increased survival, particularly in mpk-1 mutants. The effect of *R. planticola* on *P. aeruginosa* PA14 was also assessed, with no significant change in PA14 CFUs in the presence of *R. planticola* compared with OP50 (Figure 3E), suggesting that the protection offered by *R. planticola* does not involve the direct inhibition of PA14 growth. The presence of *R. planticola* significantly influenced reproductive outcomes in *C. elegans* (Figure 3F). The number of brood sizes was significantly lower in worms fed *R. planticola* than in those fed OP50, reflecting a potential trade-off between bacterial protection and long-term reproductive health. These results highlight the significant role that specific gut bacteria, particularly *R. planticola*, play in conferring resistance to *P. aeruginosa* and influencing reproductive health. However, the variability in bacterial efficacy suggests the complexity of host–microbe interactions and their impact on *C. elegans* health and survival.

### 3.5. Lack of Immune Response in let-60/mpk-1 Mutants Permits R. planticola Colonization

**Figure 4:**
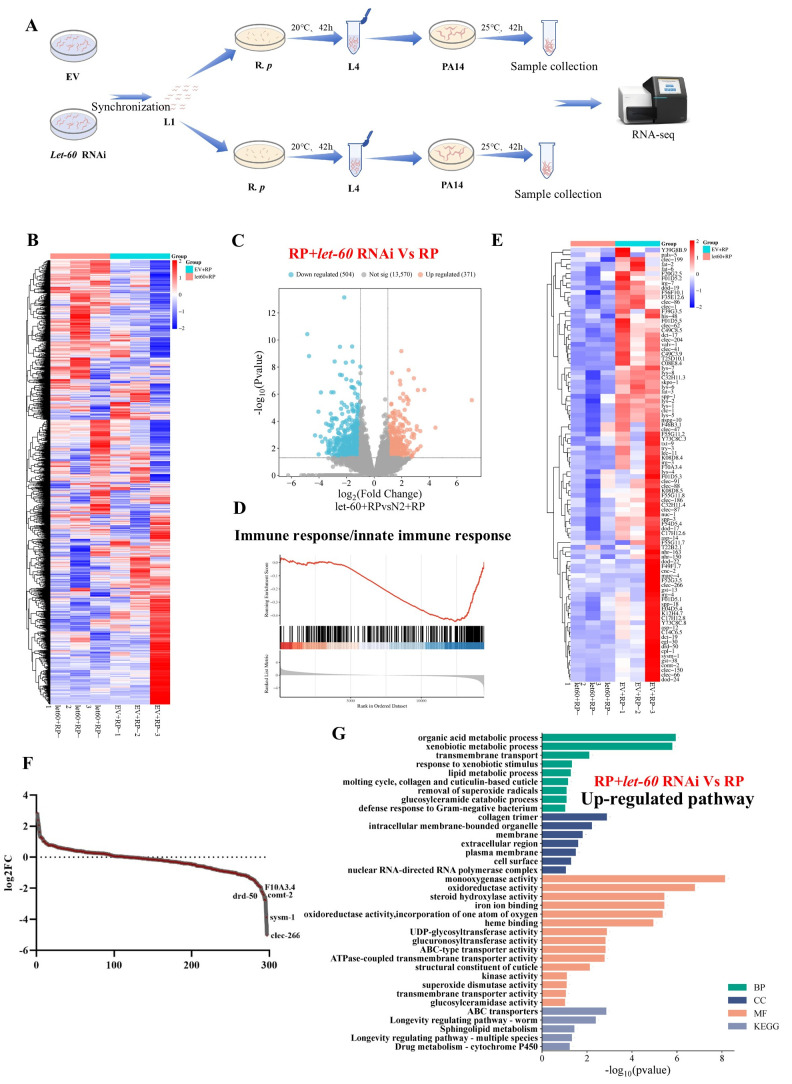
Lack of immune response in *let-60/mpk-1* mutants permits *R. planticola* colonization. (**A**) Workflow chart of the transcriptomes of the *let-60+R. planticola* and EV+*R. planticola* groups. (**B**) Heatmap of up- and downregulated genes in the *let-60+R. planticola* vs. EV+*R. planticola*. group. (**C**) Volcano plot of up- and downregulated genes in the *let-60+R. planticola* vs. EV+*R. planticola* group. (**D**) KEGG enrichment analysis suggested that *let-60* is negatively correlated with autophagy genes. (**E**) Heatmap of genes upregulated and downregulated in the *let-60+R. planticola* vs. EV+*R. planticola* group. In the heatmap, red and blue represent the genes whose expression increased and decreased, respectively, in the *R. planticola* + *let-60* RNAi group compared with the EV+*R. planticola* group. (**F**) Selection of defense response to bacteria, defense response to Gram-positive bacteria and innate immune response-related pathways for visualization among all GO-enriched pathways. (**G**) Enriched signaling pathways were visualized.

To decipher why *R. planticola* can be enriched in mpk-1 mutants, we performed transcriptomic analysis comparing changes in gene expression between N2 and those with *let-60* (a key component in ERK signaling upstream of *mpk-1*) knockdown (Figure 4A). Heatmap analysis of gene expression under the two conditions is shown in Figure 4B, revealing significant changes in gene expression between the two groups. The analysis revealed substantial upregulation of 371 genes (red dots) and downregulation of 504 genes (blue dots) (Figure 4C). Gene set enrichment analysis (GSEA) revealed that “autophagy” was enriched in the *R. planticola* group compared with the *R. planticola* + *let-60* RNAi group (Figure 4D), indicating that immune response-related genes were significantly altered in the *let-60* RNAi group (Figure 4E). A closer look at the specific gene expression changes shown in Figure 4F revealed a sharp decline in the expression of key autophagy genes (*drd-50*, *clec-266*, *sysm-1*, and *comt-2*), indicating that *let-60* RNAi suppresses immune response to facilitate *R. planticola* colonization. Further functional enrichment analysis of the down-regulated genes revealed significant pathways related to “Autophagy”, “developmentfal processes”, “defense response to bacteria” and “immune response” (Figure S4). These changes suggest that autophagy inhibition may enhance *R. planticola* colonization by modulating immune and developmental pathways in *C. elegans*. Finally, Figure 4G shows the top enriched upregulated pathways, including “longevity regulating pathway-worm” and “Drug metabolism–cytochrome P450”, suggesting that autophagy inhibition may influence longevity and metabolic processes associated with *R. planticola* colonization. Together, these results indicate that the inhibition of let-60/mpk-1-mediated autophagy promotes *R. planticola* colonization, modulates immune responses and enhances longevity-regulating pathways in *C. elegans*.

### 3.6. R. planticola Elicits Autophagy to Confer Resistance to P. aeruginosa PA14

**Figure 5:**
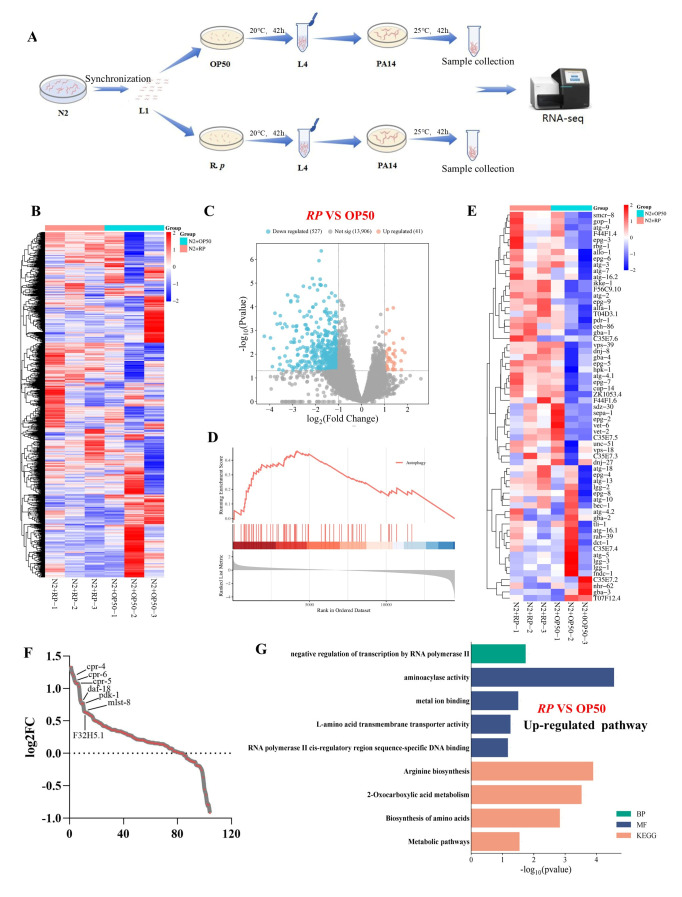
*R. planticola* elicits a different autophagy to confer resistance to PA14. (**A**) Workflow chart of the transcriptome of the *R. planticola* and OP50 groups. (**B**) Heatmap of up- and downregulated genes in the *R. planticola* and OP50 groups. (**C**) Volcano plot of up- and downregulated genes in the *R. planticola* and OP50 groups. (**D**) *R. planticola* was positively correlated with autophagy genes. (**E**) Heatmap of genes whose expression was upregulated or downregulated during autophagy in the *R. planticola* and *E. coli* OP50 groups. In the heatmap, red and blue represent the genes whose expression was upregulated and downregulated, respectively, in the *R. planticola* group compared with the *E. coli* OP50 group, and *E. coli* OP50 was negatively correlated with autophagy genes in the *R. planticola* and OP50 groups. (**F**) Genes whose expression was significantly upregulated in RP compared with that in OP50. (**G**) GO terms and KEGG pathway analysis of upregulated and downregulated genes in *R. planticola* vs. *E. coli* OP50.

To investigate how *R. planticola* influences autophagy and confers resistance to *P. aeruginosa* PA14, we conducted transcriptomic analyses comparing *R. planticola* with the control *E. coli* OP50 (Figure 5A). Heatmap analysis of DEGs between *R. planticola* and *E. coli* OP50 groups revealed notable expression changes in the presence of *R. planticola* (Figure 5B). The volcano plot revealed that 41 genes were upregulated (red dots) and 527 genes were downregulated (blue dots), indicating substantial gene expression alterations between *R. planticola* and *E. coli* OP50 (*p* < 0.05 and a fold change cutoff > 2.0) (Figure 5C). Gene set enrichment analysis (GSEA) revealed that the autophagy pathway was significantly enriched in the *R. planticola* group (Figure 5D), suggesting that *R. planticola* induces distinct autophagic processes in *C. elegans* to promote resistance to PA14. Further analysis of the gene expression changes shown in Figure 5E–G revealed specific genes, such as *cpr-4*, *pre-6*, and *dp-14*, which were upregulated in RP compared with *E. coli* OP50, indicating a potential regulatory role in autophagy and immune response pathways. Figure S5 shows the results of the functional enrichment analysis of the DEGs. Key pathways, such as “defense response to morphogenesis”, “Gram-negative bacterium”, and “innate immune response”, were significantly down-regulated, with *R. planticola* activating immune-related pathways, particularly in the context of bacterial defense. These findings suggest that *R. planticola* not only influences autophagy but also impacts the host lifespan by modulating longevity and immune-related pathways. In conclusion, *R. planticola* induces a distinct autophagic response to promote colonization in *C. elegans* and confers resistance to PA14. These effects are mediated through the activation of immune responses and the modulation of longevity pathways, highlighting the complex interplay between the gut microbiota and host physiology.

Next, we investigated whether the regulation of autophagy by the *let-60*/*mpk-1* pathway influences *R. planticola*-induced autophagy. We identified overlapping genes via a Venn diagram to compare the differential gene expression levels in the transcriptomes of *C. elegans* from two groups: *R. planticola* vs. *E. coli* OP50 and *R. planticola* + *let-60* RNAi vs. *R. planticola*. We found only two common genes between the upregulated genes in the *R. planticola* and *E. coli* OP50 groups and the downregulated genes in the *R. planticola* + *let-60* RNAi and *R. planticola* groups (Figure S6A). In contrast, 49 common genes were identified between the downregulated genes in the *R. planticola* vs. *E. coli* OP50 group and the upregulated genes in the *R. planticola* + *let-60* RNAi vs. *R. planticola* group. After these common genes were excluded, KEGG pathway enrichment analysis revealed significant enrichment in pathways related to “response to xenobiotic stimulus” and “innate immune response” (Figure S6B). These results indicate that although *R. planticola* was significantly enriched in the gut of *mpk-1* mutants, the protective effect of this bacterium against pathogens did not depend on the two key components, *mpk-1* and *let-60*, in the ERK/MPK-1 signaling pathway.

### 3.7. BEC-1 Is Essential for R. planticola-Induced Autophagy and Resistance of Worms to P. aeruginosa PA14

**Figure 6:**
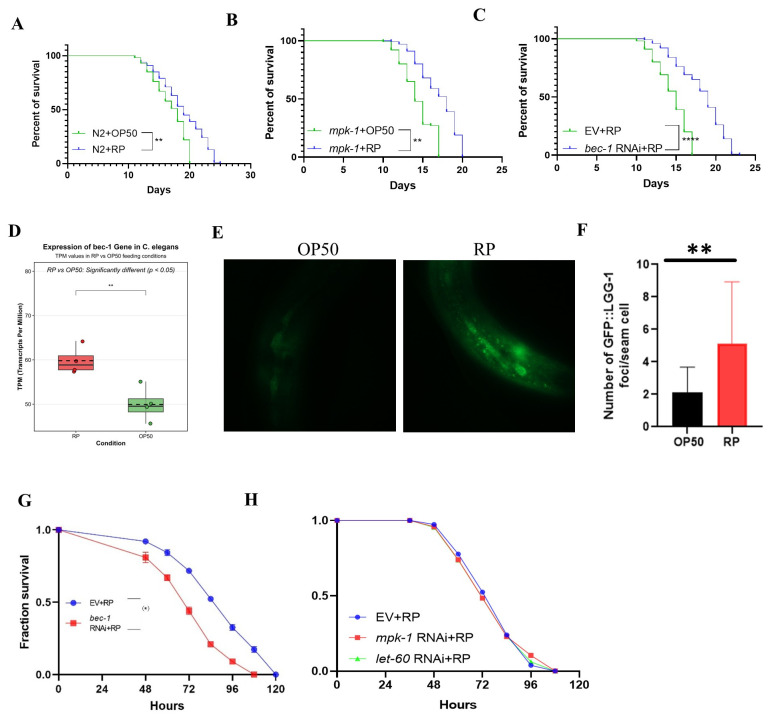
*Bec-1* is essential for *R. planticola*-induced autophagy and the resistance of worms to *PA14*. (**A**) Lifespan was determined in wild-type (N2) worms grown on *R. planticola* and *E. coli* OP50. (**B**) Lifespan was determined in *mpk-1* mutants. (**C**) *R. planticola* extends the lifespan of worms via *bec-1-*mediated autophagy. Knockdown of *bec-1* by RNAi inhibited the lifespan extension of worms growing on *R. planticola*. (**D**) TPM of bec-1 in *E. coli* OP50 or *R. planticola* in RNA-seq data. (**E**) Representative images of autophagosomes (*GFP:LGG-1* puncta) in the seam cells of worms exposed for 36 h to *E. coli* OP50 or *R. planticola*. (**F**) The number of *GFP:LGG-1* puncta was counted in the seam cells of worms. The arrow denotes a representative autophagosome. (**G**) *R. planticola* confers resistance to *P. aeruginosa* PA14 via *bec-1-*mediated autophagy in worms. (**H**) *mpk-1 or let-60* is not involved in *R. planticola*-mediated resistance to *P. aeruginosa*. The means ± SD of three separate, triplicate experiments are presented. *p* values in (**A**–**C**,**F**,**G**) were calculated using a one-way ANOVA, and in (**E**) were calculated using Student’s *t*-test. * *p* < 0.05, ** *p* < 0.01, **** *p* < 0.0001; ns, not significant.

As *R. planticola* induces autophagy, we hypothesized that, compared with *E. coli* OP50, this bacterium would extend the lifespan of worms. We used *R. planticola* and *E. coli* OP50 as dietary sources for the worms. Notably, we found that wild-type worms grown on *R. planticola* lived longer than those fed *E. coli* OP50 (Figure 6A). These data suggest that the type of bacterial food can profoundly impact the lifespan of the worms, with *R. planticola* offering a survival advantage over the standard *E. coli* OP50 diet. In a parallel experiment, we fed mpk-1 mutant worms *R. planticola* or *E. coli* OP50. We observed that mpk-1 mutants fed *R. planticola* still lived longer than those fed *E. coli* OP50 (Figure 6B). These results indicate that *R. planticola* can confer survival benefits even in the absence of functional *mpk-1*, highlighting its potential role in promoting longevity through alternative mechanisms. To test whether *R. planticola* extends the lifespan of worms via autophagy, we suppressed autophagy by silencing *bec-1* via RNAi. We found that the knockdown of *bec-1* significantly shortened the effect of *R. planticola* on the longevity of the worms (Figure 6C). These results suggest that autophagy is involved in the lifespan extension mediated by *R. planticola*. In this study, GSEA revealed significant enrichment of autophagy-related genes in *C. elegans* exposed to *R. planticola*. To test this idea, we investigated whether *R. planticola* induced autophagy in transgenic worms harboring the GFP:LGG-1 marker. The presence of GFP:LGG-1 puncta is widely recognized as a reliable indicator of autophagy. To compare autophagy levels, we fed the worms *R. planticola* or *E. coli* OP50. Through this analysis, we observed a significant increase in the number of GFP: LGG-1 puncta in the seam cells of worms fed *R. planticola* compared with those in the seam cells of worms fed *E. coli* OP50. This marked increase in autophagy suggested that the *R. planticola* strain induced increased levels of autophagy in the worms (Figure 6D,E).

To test whether *R. planticola* confers resistance to *P. aeruginosa* PA14 via autophagy, we suppressed autophagy by silencing *bec-1* (the *C. elegans* ortholog of ATG6/VPS30/beclin1), a key autophagy-related gene, via RNAi in worms. We found that the knockdown of *bec-1* significantly inhibited the protective effect of *R. planticola* on resistance to *P. aeruginosa* PA14 in the worms (Figure 6F). These results suggest that autophagy is required for host defense against the pathogen mediated by *R. planticola*. As mentioned above, *R. planticola* was most abundant in the *mpk-1* mutants. We thus examined whether *mpk-1* or *let-60*, the key components in ERK signaling, play a functional role in host defense against the pathogenic bacteria *P. aeruginosa* PA14 triggered by *R. planticola*. We performed RNAi to knock down these genes and then tracked their survival over time. Our results revealed that the survival rate of *R. planticola*-treated worms subjected to either *mpk-1* or *let-60* RNAi was similar to that of those subjected to the empty vector (EV) after *P. aeruginosa* PA14 infection (Figure 6G). These results suggest that *R. planticola* confers resistance to *P. aeruginosa* PA14 in worms independent of *mpk-1* or *let-60*.

## 4. Discussion and Conclusions

Microorganisms have coexisted with life forms for billions of years, developing intricate and symbiotic relationships with plants, animals, and humans. These microbes, which are abundant and genetically diverse, play critical roles in host physiology, particularly through the gut microbiota, which influences traits such as reproduction, aging, and metabolism [13]. While extensive research has focused on the composition and effects of the gut microbiota, questions remain about its dynamics across generations, inheritance, and resistance to pathogens. *C. elegans* serves as an excellent model for studying host–microbe interactions because of its interactions with a variety of microbes, including pathogens [14]. Although its gut microbiota plays a role in defense and mutualism, longitudinal studies linking microbiota dynamics with host traits and vertical transmission are limited. In this study, *C. elegans* was used to explore how host genetic factors shape gut microbial communities and how microbiota influence resistance of the host to pathogens. Using wild-type worms (N2) and five mutants (*mpk-1*, *egl-44*, *hlh-30*, *atfs-1*, *ced-3*), we identified distinct gut microbiomes associated with different genetic backgrounds, highlighting the significant effect of host genetics on microbial composition. Only 20 ASVs were common to all groups, and 144 ASVs were shared across all worm groups (N2 strain and five mutants), while a significant number of unique ASVs were observed in the individual mutant strains (*mpk-1*, *egl-44*, and *ced-3*). Because these mutants are immune-compromised, community differences likely reflect a combination of genotype-specific effects and increased colonization permissiveness; accordingly, we interpret microbiome shifts cautiously in terms of mechanism. Further investigation revealed that impairment of *let-60/mpk-1*-linked programs increased permissiveness to *R. planticola* enrichment/colonization, whereas *R. planticola* exposure improved survival during PA14 infection and extended lifespan through *bec-1*–dependent autophagy, and this protective effect did not require intact *let-60/mpk-1* signaling.

Microbial survival and growth in the human host are shaped by evolutionary adaptations that enable pathogens to thrive. For example, *Salmonella enterica serovar Typhimurium* (*S. Tm*) and *Shigella* spp. have evolved to establish long-term, microbe-favorable relationships with humans, potentially facilitating their transmission. Mechanistically, *S. Tm* colonization triggers the innate immune response, generating reactive nitrogen species, which the pathogen utilizes for anaerobic respiration, thereby gaining a fitness advantage over other microbes. Additionally, host genetic variation influences susceptibility to enteric diseases, as demonstrated by a genome-wide association study on Shigella-induced diarrhea in Bangladeshi infants, which identified both protective and risk-associated loci [15]. This study employed *C. elegans* to explore the impact of host genetics on the gut microbiota and the influence of gut bacteria on host traits. While most *C. elegans* studies use *E. coli* OP50, natural nematodes inhabit soil environments rich in diverse microbial communities. We raised wild-type worms (N2) and five immune-related gene mutants (*mpk-1*, *atfs-1*, *egl-44*, *ced-3*, and *hlh-30*) in soil and identified a total of 25 phyla, 49 classes, 123 orders, 227 families, and 533 genera through full-length 16S rRNA sequencing. The most abundant phyla included *Proteobacteria*, *Firmicutes*, *Bacteroidota*, and *Actinobacteriota*, with *R. planticola* significantly enriched in mpk-1 mutants, suggesting its potential role as a biomarker. *Gammaproteobacteria* dominated the microbiota across the soil, N2, and mutant samples, with variations in relative abundance reflecting genetic differences. Our findings demonstrate that host genetics significantly shape gut microbial communities and highlight the presence of a core microbiota in *C. elegans*. Despite the well-documented influence of the gut microbiota on host traits such as resistance to pathogens, longitudinal studies exploring the relationships between microbiota dynamics and host genetics are lacking. This study addresses this gap, providing valuable insights into the regulatory mechanisms of host–microbiota interactions. Given the frequent interactions of *C. elegans* with diverse microbes and its robust defense systems, it remains an invaluable model for studying the dynamic and mutualistic relationships between hosts and their microbiota.

The benefits microbiomes provide to their hosts have garnered considerable attention, with growing evidence linking these benefits to mechanisms of host control. Over hundreds of millions of years, natural selection has driven multicellular hosts to develop sophisticated adaptations to manage diverse and highly adaptable microbial communities, creating a delicate balance between host regulation and microbial evolution. This ongoing tension offers a framework to better understand microbiome biology and its implications for health and disease. Host control mechanisms over complex microbiotas, such as the ability to selectively favor beneficial microbes, highlight potential therapeutic targets for reshaping the microbiome to promote health. Recent advancements, including larger genome-wide association studies (GWASs), have identified genetic loci (e.g., LCT, ABO) that link specific genes, dietary habits, and secretor status to microbial taxa and metabolic pathways [16]. In this study, we found that the enrichment of *R. planticola* in mpk-1 mutants resulting from autophagy inhibition promoted resistance to *P. aeruginosa* PA14 and extended the nematode lifespan by activating bec-1-mediated autophagy. These findings provide experimental evidence supporting the notion that host genetic factors influence bacterial diversity in the gut of *C. elegans*, shaping host innate immunity. However, these results emphasize the need for even larger sample sizes to fully elucidate the effects of host genetics on the gut microbiome. Variability in results across studies often arises from differences in sample sizes, technical and analytical methods, population diversity, and adjustments for clinical metadata, as well as the challenges posed by cross-sectional sampling in systems known for their temporal dynamics. Current evidence suggests that heritable microbial taxa exhibit limited temporal stability, complicating efforts to draw definitive conclusions. Addressing these challenges will require harmonizing methodologies, expanding datasets, and considering the dynamic nature of microbiomes over time. By doing so, we can pave the way for a deeper understanding of how host genetics influence microbiome structure and function, ultimately enabling the development of targeted strategies to optimize host–microbe interactions for improved health outcomes.

Our findings align with a broader shift in host–microbiota research from a predominantly “microbe-to-host” perspective toward increasing recognition that host genetic variation can shape microbiota composition. Large-scale human studies have identified reproducible single nucleotide polymorphism (SNP)-taxon associations (including loci such as LCT (lactase-phlorizin hydrolase) and ABO glycosyltransferase), demonstrating that host genotype—often interacting with environment and diet—contributes to differences in gut microbial abundance patterns. Mechanistic studies in *C. elegans* likewise show that disruption of conserved immune pathways (e.g., the TGFβ/BMP signaling) can drive blooms of specific commensals and alter spatial control of colonization, highlighting pathway-dependent gene–microbe specificity. Together with these studies, our work supports the view that host genetics can actively shape microbial homeostasis, and colonizing bacteria can feed back on host defense and life-history traits.

A key factor in maintaining the health span of *C. elegans* is the innate immune system, which is governed by several signaling pathways, including ERK MAPK MPK-1, ATFS-1, EGL-44, CED-3, and HLH-30 [17]. To investigate how host genetics impact the gut microbiota, we selected five different mutants (*mpk-1*, *atfs-1*, *egl-44*, *ced-3*, and *hlh-30*), all of which are considered immune-deficient worms. Autophagy plays a crucial role in extending lifespan, as demonstrated in studies with *C. elegans daf-2* mutants and other longevity-associated mutations, where autophagy was consistently shown to be essential. Since the initial discovery of the role of autophagy in lifespan extension, various mutations leading to increased longevity have been identified, consistently indicating that autophagy is necessary. Methods that elicit autophagy, such as dietary restriction (including caloric restriction, reduced food intake, nutrient limitations, and intermittent fasting), also promote lifespan extension, with the *eat-2* mutant serving as a dietary restriction model owing to its reduced food intake and extended lifespan. Our study revealed that *R. planticola* triggers bec-1-mediated autophagy as a defensive mechanism, helping worms defend against *P. aeruginosa* PA14 infection and extending their lifespan. This provides a direct link between autophagy and immune defense. In addition to inducing autophagy, *R. planticola* upregulates lysozyme genes such as *lys-1*, *lys-2*, *lys-7*, and *lys-8* to enhance immune responses and pathogen defense, which is consistent with previous reports. For example, *clec-41* encodes an antimicrobial protein critical for defense against Bacillus thuringiensis, whereas *lys-1* and *lys-2* contribute to resistance against *Serratia marcescens* and *B. thuringiensis*, respectively [11]. *lys-7* and *lys-8* are crucial for maintaining a normal lifespan by limiting gut bacterial accumulation, highlighting their roles in immunity and aging [18,19]. These findings suggest that autophagy and immune gene regulation might be key mechanisms linking microbial interactions to host longevity and health in our study.

Our findings demonstrate that *R. planticola* significantly reduces brood size in *C. elegans* while simultaneously extending lifespan, likely through its ability to increase autophagic activity in the host. This suggests a trade-off between fecundity and lifespan, where the type of bacterial diet profoundly influences worm survival and reproductive strategies. These dietary effects highlight how bacterial food sources can optimize specific host traits, offering advantages over the standard *E. coli* OP50 diet. For example, *R. planticola* has been shown to improve *C. elegans* survival following treatment with the cardiotoxic anticancer drug doxorubicin, indicating its potential as a therapeutic probiotic. Bacterial diets are known to modulate the lifespan and health span of *C. elegans*, with certain strains, such as *Lactobacillus*, demonstrating mild benefits in promoting both longevity and overall health [20]. These results underscore the importance of the bacterial diet as a key determinant of host life history traits and suggest that manipulating bacterial food sources could provide valuable insights into the mechanisms linking diet, metabolism, and aging. The observed reduction in brood size under *R. planticola* feeding/colonization represents an important fitness cost and should be interpreted in the context of life-history theory. Trade-offs between survival and reproduction are widespread, and in *C. elegans*, reproductive output is coupled to nutrient sensing, stress responses, and cellular homeostasis programs that also influence survival. Reduced brood size may therefore reflect a resource-allocation shift toward maintenance and defense when worms interact with *R. planticola*, consistent with the parallel increase in pathogen resistance and longevity. This pattern is also compatible with conditional mutualism: a commensal can be beneficial under pathogen pressure (enhanced survival during *P. aeruginosa* PA14 infection) while imposing a cost in pathogen-free settings (reduced fecundity). Because we did not directly quantify developmental timing or metabolic status, we cannot exclude the possibility that brood-size reduction is partially secondary to altered development or metabolism induced by the bacterial diet; stage-matched reproduction assays and metabolic profiling will be useful in future work.

Several limitations should be acknowledged. First, 16S rRNA sequencing captures community composition but provides limited strain-level resolution and does not directly quantify functional potential; shotgun metagenomics would better define microbial genes/pathways, and metabolomic profiling may help clarify how signature microbes, in turn, influence host functions. Second, community profiling provides associative signals and cannot alone establish causality; additional gnotobiotic reconstructions and competition experiments will strengthen causal inference. Third, soil-derived communities are heterogeneous across preparations; although we standardized handling and assessed technical effects, residual batch and growth-related biases may influence observed enrichment. Fourth, while five mutant backgrounds were profiled, mechanistic follow-up focused primarily on *mpk-1*, limiting generalizability; systematic validation across additional host genotypes will be needed. Finally, not all transcriptome-implicated genes were functionally tested, so enrichment results are treated as hypothesis-generating and mechanistic conclusions are restricted to relationships supported by functional assays (e.g., *bec-1* dependence).

In conclusion, we found that *R. planticola* is enriched in *mpk-1* mutants and *let-60*-silenced worms because of attenuated immune response and autophagy. The enrichment of *R. planticola* promotes nematode resistance to *P. aeruginosa* PA14 infection and extends the lifespan of *mpk-1* mutants via *bec-1*-mediated autophagy. We therefore propose a two-step framework in which host genotype shapes colonization permissiveness, and colonizing bacteria modulate host defense and longevity through autophagy. Our study highlights how host genetic factors influence bacterial communities in the gut of *C. elegans* and how the intestinal microbiota affects *C. elegans* health and immunity. This provides an in-depth exploration of the regulatory mechanisms underlying host–microbiome interactions from this perspective.

## Data Availability

The datasets produced in this study are available in the following databases: The RNA-Seq data generated in this study have been deposited in the SRA database; 16S rRNA sequencing data have been deposited in the SRA database (PRJNA307527).

## Supplementary Materials

The following supporting information can be downloaded at: https://www.mdpi.com/article/10.3390/microorganisms14030551/s1, Figures S1–S6: Supplementary Figure S1. Microbiome compositions of soil, N2, and the five mutants at different levels; Supplementary Figure S2. Phylogenetic tree of gut cultured bacteria and their abundance in N2 and different mutants; Supplementary Figure S3. Indicator species analysis of different groups of soil, the N2 strain and the mpk-1, egl-44, atfs-1, ced-3 and hlh-30 mutants; Supplementary Figure S4. *let-60 deficient promotes R. planticola colonization*; Supplementary Figure S5. GO terms and KEGG pathway analysis of downregulated genes in *R. planticola* vs. *E. coli* OP50; Supplementary Figure 6. *let-60/mpk-1 deficient reduced immune response does not affect bec-1 mediated autophagy*; Tables S1–S4: Supplementary Table S1. *C. elegans* strains used in the experiment; Supplementary Table S2. Bacterial strains used in the experiments; Supplementary Table S3. Experimental reagents; Supplementary Table S4. PCR primers used in the experiment.
