# Supplementary material for: Genetic Control of Gut Microbial Diversity Enhances Host Resistance to Pathogenic Infections in C. elegans
Source: Microorganisms. 2026 Feb 27;14(3):551. doi: 10.3390/microorganisms14030551 (PMC13029074; doi:10.3390/microorganisms14030551)
Supplement: Supplementary file 1 [file microorganisms-14-00551-s001.zip › Supplementary Figures.pdf]

Article

Genetic Control of Gut Microbial Diversity Enhances Host Resistance to Pathogenic Infections in *C. elegans*

Rahat Ullah Khan [1,†], Boyang Zhang [1,†], Hengcheng Liu [1], Wenping Wu [1], Jianqi Yang [2], Yi-Cheng Ma [1], Cheng-Gang Zou [1,3,*] and Ping Jin [1,*]

Supplementary Figures

Figure S1. Microbiome composition of Soil, N2, and the five mutants at different levels.

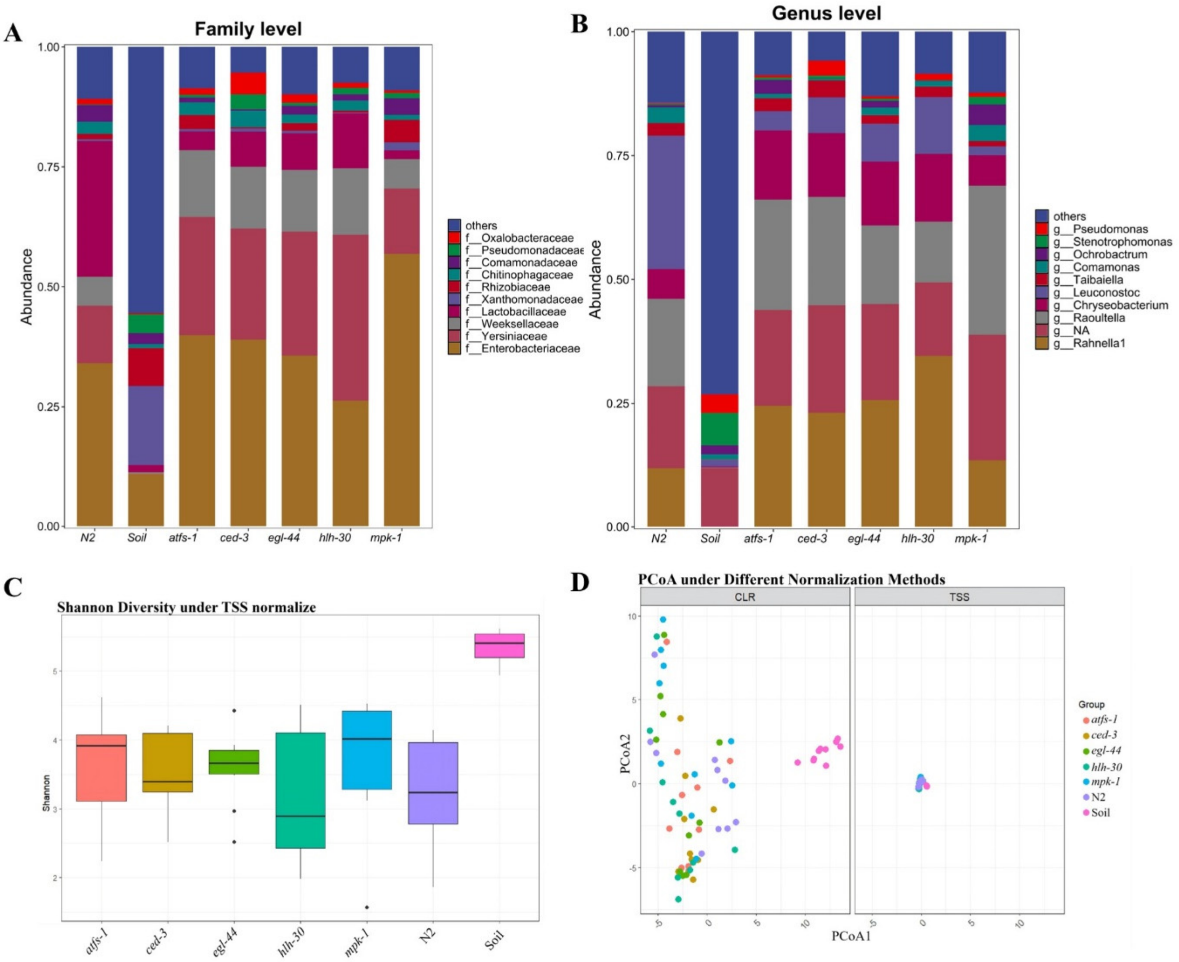

Supplementary Figure S1. Microbiome compositions of soil, N2, and the five mutants at different levels. (A-B) The top ten species in terms of abundance are shown in the bar plots; the others are combined as “others” in different groups of soil, the N2 strain and the *mpk-1*, *egl-44*, *atfs-1*, *ced-3* and *hlh-30* mutants. **(A)** Bacterial composition at the family level. **(B)** Bacterial composition at the genus level. **(C)** Shannon diversity under Total Sum Scaling (TSS) normalize. **(D)** PCoA under different normalization methods.

Figure S2. Phylogenetic tree of gut cultured bacteria and their abundance in N2 and different mutants.

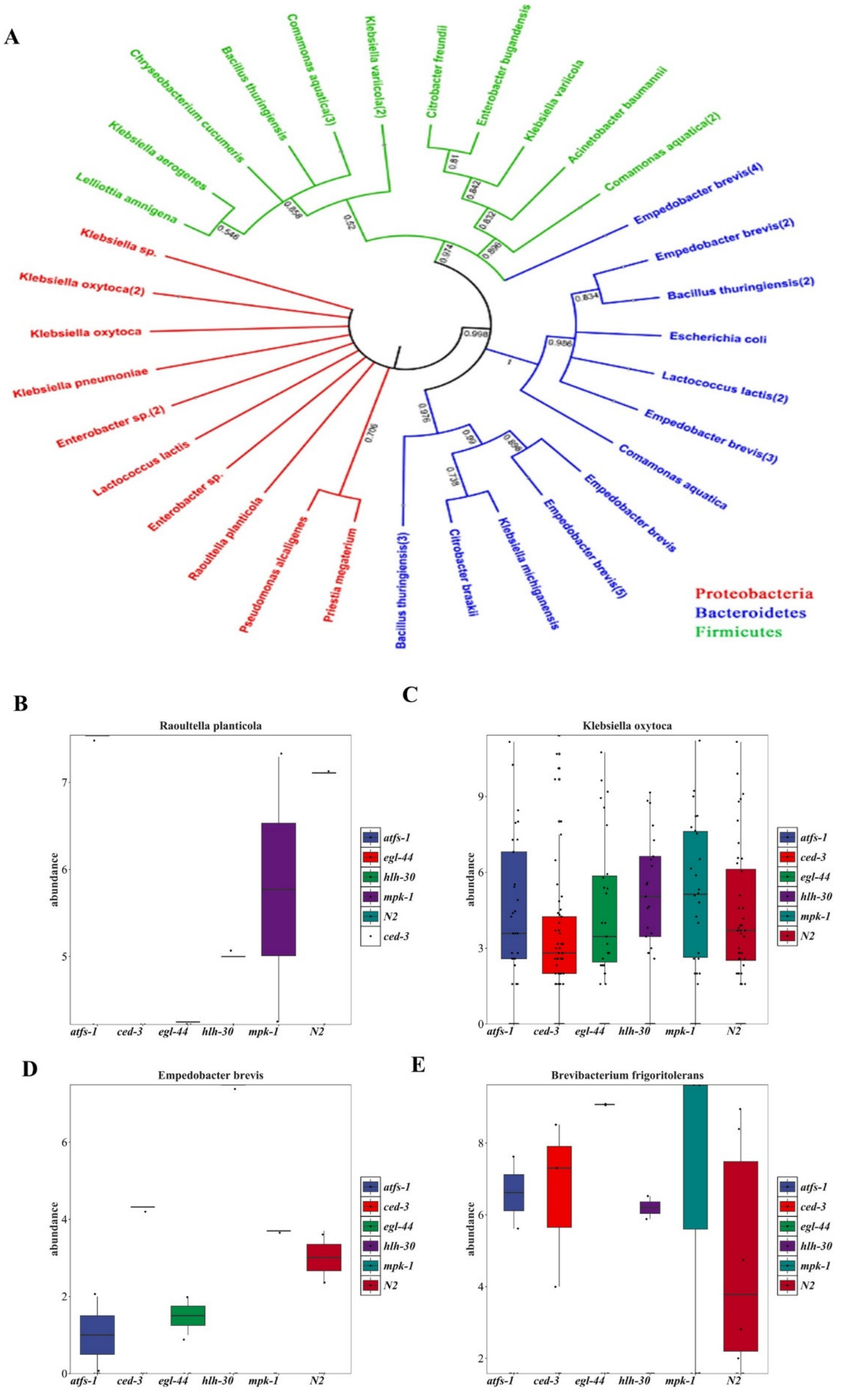

Supplementary Figure S2. Phylogenetic tree of gut cultured bacteria and their abundance in N2 and different mutants. (A) Phylogenetic tree of cultured gut bacteria. **(B)** Abundance of *R.planticola* in the N2 strain and these mutants. **(C)** Abundance of *Klebsiella oxytoca* in the N2 strain and these mutants. **(D)** Abundance of *Empedobacter brevis* in the N2 strain and these mutants. **(E)** Abundance of *Brevibacterium frigoritolerans* in the N2 strain and these mutants. The points in the figure represent the number of different ASV annotated to the same species (n=10).

Figure S3. *Raoultella planticola* as a biomarker in *mpk-1* mutants.

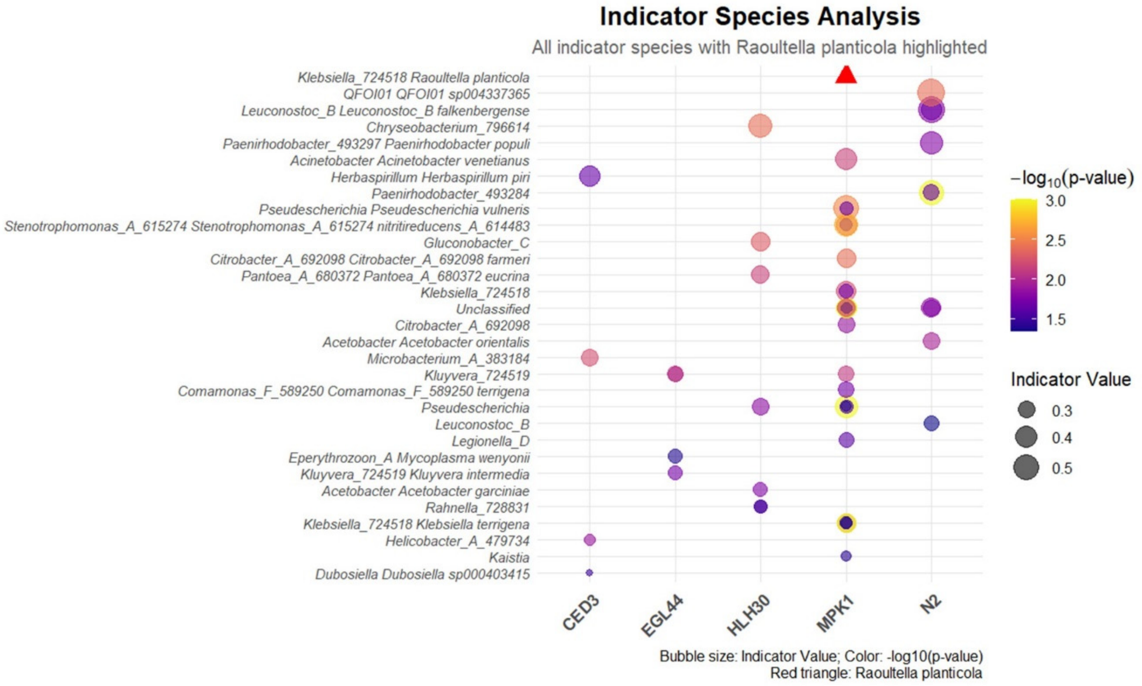

Supplementary Figure S3. Indicator species analysis of different groups of soil, the N2 strain and the mpk-1, egl-44, atfs-1, ced-3 and hlh-30 mutants.

Figure S4. *let-60* deficient promotes *R. planticola* colonization.

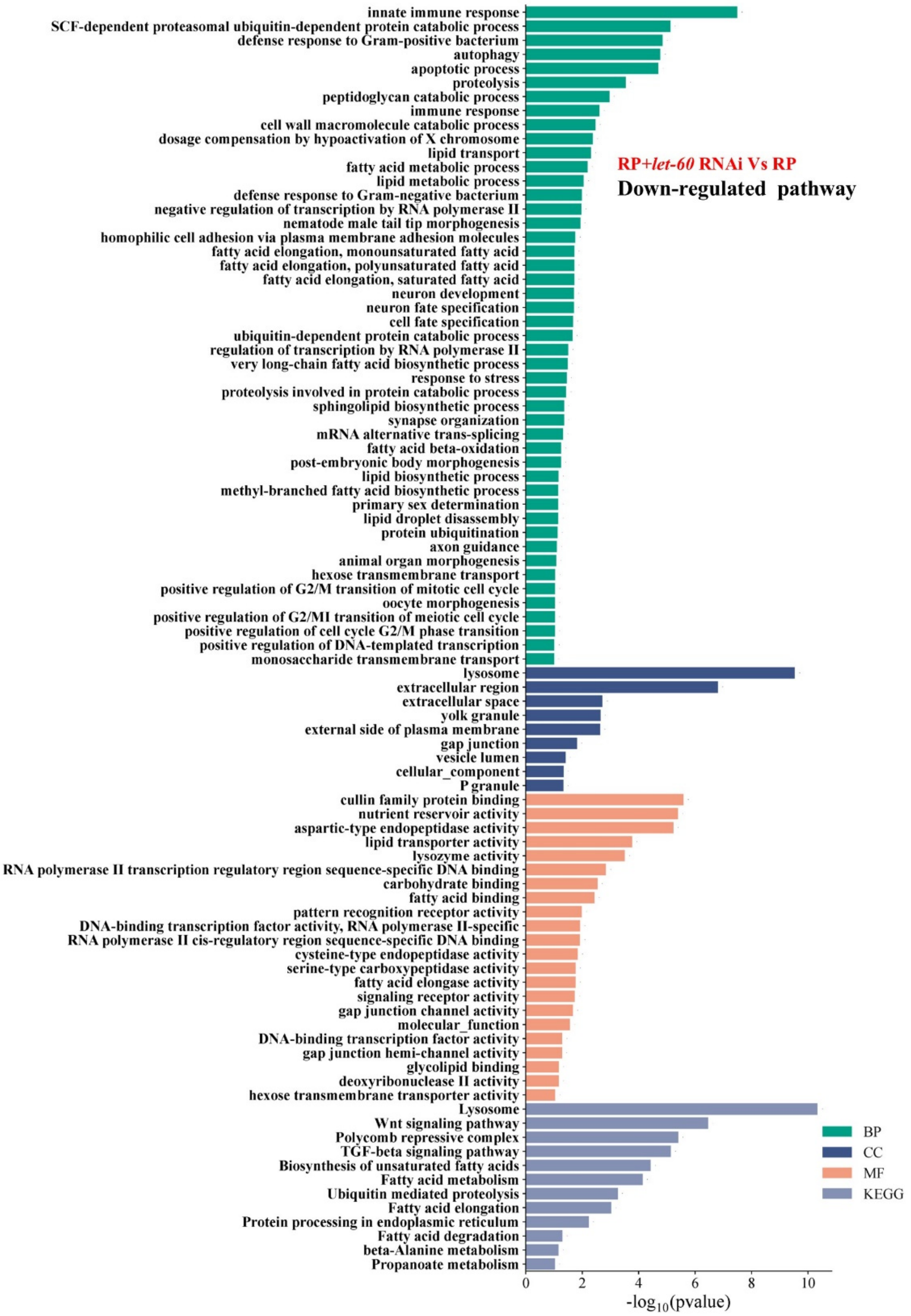

Supplementary Figure S4. *let-60 deficient promotes R. planticola colonization*. GO terms and KEGG pathway analysis of downregulated genes in *R. planticola +let-60 RNAi vs. R. planticola*. excluding three genes whose expression overlapped with that of *R. planticola* +*let-60* RNAi vs. *R. planticola*-downregulated genes.

Figure S5. ***R. planticola*** **elicits autophagy to confer resistance to** ***PA14.***

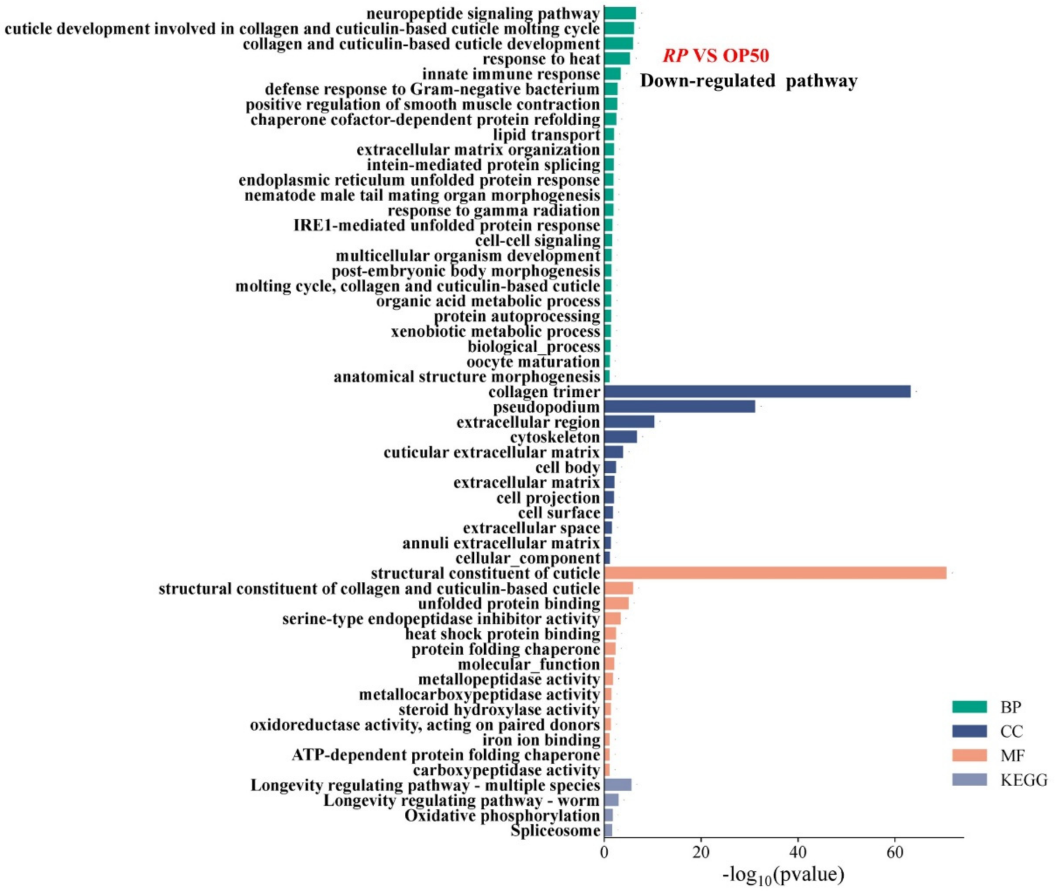

Supplementary Figure S5. GO terms and KEGG pathway analysis of downregulated genes in *R. planticola* vs. *E. coli* OP50.

Figure S6. ***let-60/mpk-1*** **deficient reduced immune response does not affect** ***bec-1 mediated*** **autophagy.**

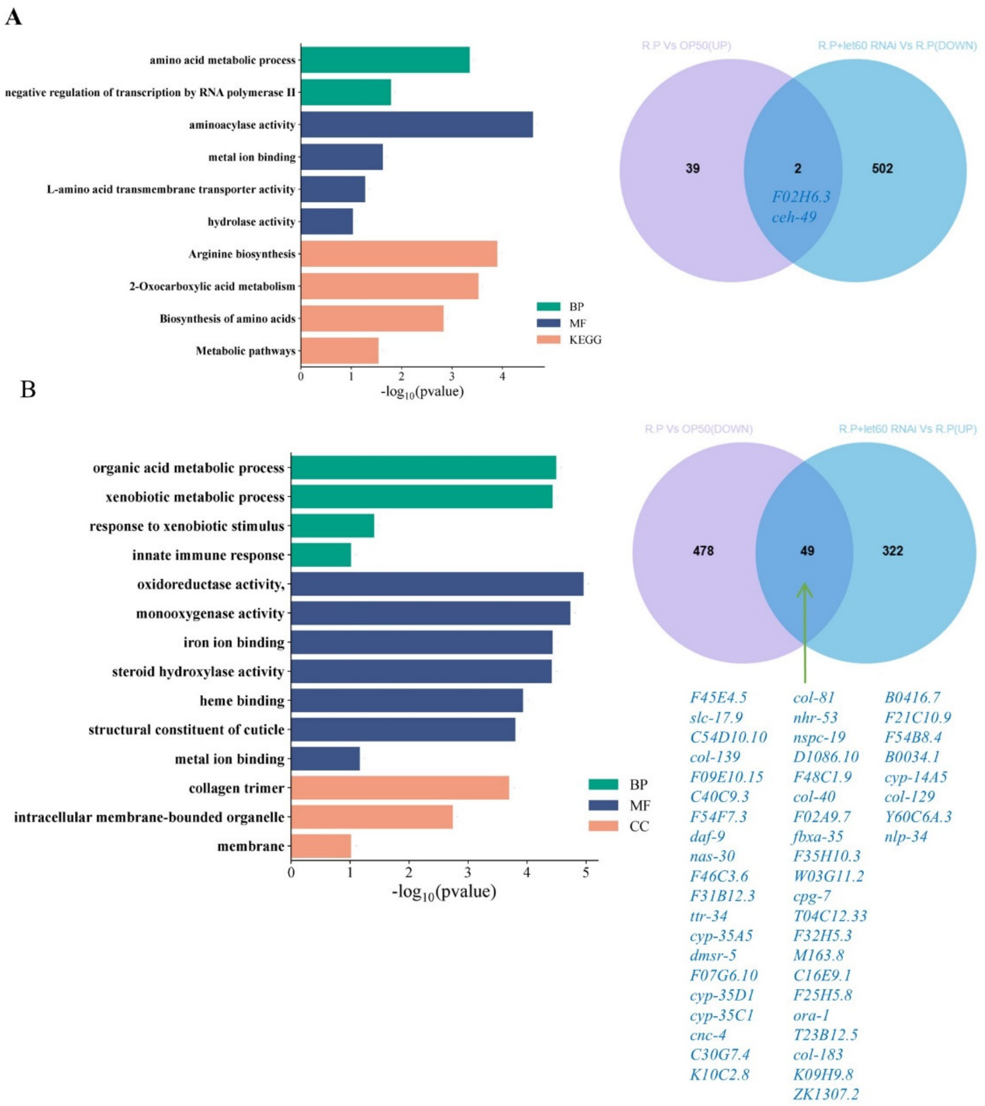

Supplementary Figure S6. ***let-60/mpk-1 deficient reduced immune response does not affect bec-1 mediated autophagy*****.** **(A)** GO terms and KEGG pathway analysis of upregulated genes in *R. planticola* vs. *E. coli* OP50, excluding two genes whose expression overlapped with that of *R. planticola +let-60* RNAi vs. *R. planticola*-downregulated genes. **(B)** GO terms and KEGG pathway analysis of 478 downregulated genes in *R. planticola* vs. *E. coli* OP50, excluding 49 genes whose expression overlapped with that of *R. planticola +let-60* RNAi vs. *R. planticola* upregulated genes.
