# Supplementary material for: Genetic Control of Gut Microbial Diversity Enhances Host Resistance to Pathogenic Infections in C. elegans
Source: Microorganisms. 2026 Feb 27;14(3):551. doi: 10.3390/microorganisms14030551 (PMC13029074; doi:10.3390/microorganisms14030551)
Supplement: Supplementary file 1 [file microorganisms-14-00551-s001.zip › Supplementary Tables.pdf]

Article

Genetic Control of Gut Microbial Diversity Enhances Host Resistance to Pathogenic Infections in *C. elegans*

Rahat Ullah Khan [1,†], Boyang Zhang [1,†], Hengcheng Liu [1], Wenping Wu [1], Jianqi Yang [2], Yi-Cheng Ma [1], Cheng-Gang Zou [1,3,*] and Ping Jin [1,*]

Supplementary Tables

Supplementary Table S1. *C. elegans* strains used in the experiment

Strain	Genotype	Source
N2 Bristol	wild type	CGC
SD184	*unc-79(e1068) mpk-1(n2521)*	CGC
DA2123	*adIs2122 [lgg-1p::GFP::lgg-1 + rol-6(su1006)]*	CGC
VC3201	*atfs-1(gk3094)*	CGC
MT1522	*ced-3(n717)*	CGC
JIN1375	*hlh-30(tm1978)*	CGC
MT2247	*egl-44(n1080)*	CGC

Supplementary Table S2. Bacterial strains used in the experiments

**Name**	**Source**
Raultella planticola	cultured from gut
mpk-1 RNAi	ahringer RNAI library
let-60 RNAi	ahringer RNAI library
E. coli OP50	ahringer RNAI library
Exiguobacterium acetylicum	cultured from gut
Klebsiella oxytoca A07	cultured from gut
Empedobacter brevis	cultured from gut
Brevibacterium frigoritolerans	cultured from gut
Klebsiella oxytoca	cultured from gut
Citrobacter telavivum	cultured from gut

Supplementary Table S3. Experimental reagents

Name of Reagent	Manufacturer	City, Country

Sodium chloride	Tianjin Wind Ship	Tianjin, China
Tryptone	Sangon Biotech (Shanghai) Co., Ltd.	Shanghai, China
Peptone	Sangon Biotech (Shanghai) Co., Ltd.	Shanghai, China
Yeast Extract	Tianjin Wind Ship	Tianjin, China
Ampicillin	McLean	Illinois, USA
Kanamycin	McLean	Illinois, USA
Levamisole	Sigma-Aldrich	St. Louis, Missouri, USA
BHI (Brain Heart Infusion)	CycloKai Biotechnology Co., Ltd.	Guangdong, China
Glucose	Sangon Biotech (Shanghai) Co., Ltd.	Shanghai, China
Beef extract	Sangon Biotech (Shanghai) Co., Ltd.	Shanghai, China
Sodium hypochlorite solution	Sangon Biotech (Shanghai) Co., Ltd.	Shanghai, China
Ethanol	Longxi Chemical	Nanjing, Jiangsu, China
Sodium hydroxide	Sangon Biotech (Shanghai) Co., Ltd.	Shanghai, China
Cholesterol	Sigma-Aldrich	St. Louis, Missouri, USA
Agar	Sangon Biotech (Shanghai) Co., Ltd.	Shanghai, China
Glycerinum	Beijing Soleil	Beijing, China
Mix enzyme for PCR	Beijing Total Gold	Beijing, China
Calcium chloride	Retrocession of Tianjin	Tianjin, China
Sulphate	Retrocession of Tianjin	Tianjin, China
FUDR	Sigma-Aldrich	St. Louis, Missouri, USA

Potassium hydrogen phosphate anhydrous	Sangon Biotech (Shanghai) Co., Ltd.	Shanghai, China
Potassium Phosphate Monobasic	Sangon Biotech (Shanghai) Co., Ltd.	Shanghai, China
Tris-base	Sangon Biotech (Shanghai) Co., Ltd.	Shanghai, China
Boric acid	Sangon Biotech (Shanghai) Co., Ltd.	Shanghai, China
Proteinase K	Sigma-Aldrich	St. Louis, Missouri, USA
Nuclease	Sigma-Aldrich	St. Louis, Missouri, USA
Tris-HCl	Sigma-Aldrich	St. Louis, Missouri, USA
Agarose	Sangon Biotech (Shanghai) Co., Ltd.	Shanghai, China
Nucleic acid dye	Sangon Biotech (Shanghai) Co., Ltd.	Shanghai, China
Disodium EDTA	Beijing Soleil	Beijing, China
Glycine	Sangon Biotech (Shanghai) Co., Ltd.	Shanghai, China
2000 bp DNA marker	Beijing Soleil	Beijing, China

Supplementary Table S4. PCR primers used in the experiment

PrimerName	sequence (5' to3')
16S-27F	AGAGTTTGATCCTGGCTCAG
16S-1492R	TACGGCTACCTTGTTACGACTT
16S-27F	AGRGTTYGATYMTGGCTCAG
16S-1492R	RGYTACCTTGTTACGACTT
